# Association of *MUC16* Mutation With Response to Immune Checkpoint Inhibitors in Solid Tumors

**DOI:** 10.1001/jamanetworkopen.2020.13201

**Published:** 2020-08-26

**Authors:** Lei Zhang, Xiaohong Han, Yuankai Shi

**Affiliations:** 1 Beijing Key Laboratory of Clinical Study on Anticancer Molecular Targeted Drugs, National Cancer Center, National Clinical Research Center for Cancer, Cancer Hospital, Department of Medical Oncology, Chinese Academy of Medical Sciences & Peking Union Medical College, Beijing, China; 2 Clinical Pharmacology Research Center, Peking Union Medical College Hospital, Chinese Academy of Medical Sciences & Peking Union Medical College, Beijing, China

## Abstract

**Importance:**

As the third most frequently mutated gene in cancers, the association between *MUC16* mutation and response to immune checkpoint inhibitors (ICIs) in solid tumors remains unclear.

**Objective:**

To examine whether *MUC16* mutation is associated with genomic factors in ICI response in solid tumors and with outcomes in ICI-treated patients.

**Design, Setting, and Participants:**

This cohort study used multidimensional genomic data of 10 195 patients from The Cancer Genome Atlas (TCGA) across 30 solid tumor types, 56 patients from a non–small cell lung cancer (NSCLC) cohort, and 145 patients from a melanoma cohort. Genomic factors associated with ICI response covered tumor mutational burden, neoantigens, immune-related gene signatures, and tumor immune microenvironment. Both NSCLC and melanoma cohorts included ICI-treated patients. The TCGA cohort was used to examine the association of *MUC16* mutation with genomic factors. Two ICI-treated cohorts were used to explore the significance of outcomes associated with *MUC16* mutation, using Kaplan-Meier curves and Cox models with adjusting for potential confounders. Gene set enrichment analysis was used to identify *MUC16* mutation–associated biological processes. Data were obtained from October 1 through October 10, 2019, and were analyzed from October 11 through December 31, 2019.

**Main Outcomes and Measures:**

Genomic factors associated with ICI response, overall survival, and clinical response.

**Results:**

Of the 10 195 patients, 4821 (47.6%) were men (median [interquartile range {IQR}] age, 60 [50-70] years). *MUC16* was mutated in 2006 of 10 195 patients (19.68%). In this pan-cancer data set, patients with *MUC16* mutation had higher tumor mutational burden (median [IQR], 230 [93-595] mutations vs 48 [25-92] mutations; difference, 182 mutations; 95% CI, 164-199 mutations; *P* < .001) and neoantigen load (median [IQR], 179 [74-394.5] neoantigens vs 48 [24-89] neoantigens; difference, 131 antigens; 95% CI, 116.5-145 neoantigens; *P* < .001) than those without mutations. The tumor immune microenvironment with dual-positive *CD8A* and *PD-L1* was overrepresented in *MUC16*-mutated tumors compared with wild-type ones (43.8% vs 32.4%; odds ratio, 1.63; 95% CI, 1.46-1.80; *P* < .001). Of the 40 immune-related genes, 37 (92.5%) exhibited differential expression between 2 states. *MUC16* mutation was associated with improved overall survival in both the NSCLC (hazard ratio, 0.34; 95% CI, 0.12-0.99; *P* = .04) and melanoma (hazard ratio, 0.57; 95% CI, 0.36-0.90; *P* = .02) cohorts. The improvement persisted after adjusting for age, sex, and dominant mutational signatures in the melanoma cohort (hazard ratio, 0.57; 95% CI, 0.33-0.96; *P* = .04). *MUC16* mutation was associated with greater response rates in the NSCLC cohort (odds ratio, 4.03; 95% CI, 1.06-16.43; *P* = .03) and the melanoma cohort (odds ratio, 3.38; 95% CI, 1.07-14.25; *P* = .03). Gene set enrichment analysis revealed that gene sets regarding cell proliferation and immune response were enriched in *MUC16*-mutated tumors (false discovery rate, <.001).

**Conclusions and Relevance:**

*MUC16* mutation appears to be associated with reported genomic factors associated with response to and improved outcomes for ICI treatment in solid tumors. It may hold promise as a marker for guiding immunotherapeutic responsiveness.

## Key Points

**Question:**

What is the association between *MUC16* mutation and response to immune checkpoint inhibitors (ICIs) in solid tumors?

**Findings:**

In this cohort study of 3 groups of patients, including The Cancer Genome Atlas cohort with 10 195 patients across 30 solid tumor types, 56 patients with non–small cell lung cancer, and 145 patients with melanoma, *MUC16* mutation was associated with greater response rates, prolonged overall survival, and genomic factors associated with ICI response, including tumor mutational burden and programmed cell death ligand–1 expression.

**Meaning:**

These findings suggest that *MUC16* mutation may be associated with superior response to ICIs in patients with solid tumors.

## Teaser

This cohort study examines whether *MUC16* mutation is associated with response to treatment with immune checkpoint inhibitors and outcomes among patients with solid tumors, non–small cell lung cancer, and melanoma.

## Introduction

Immune checkpoint inhibitor (ICI)–based immunotherapy, which mainly targets cytotoxic T lymphocyte–associated protein 4 (CTLA-4), programmed cell death 1 (PD-1), and its ligand (PD-L1), has shown remarkable clinical benefits to patients with advanced-stage cancers.^1^ Early studies^2^ suggest that ICI response may be associated with PD-L1 protein levels, tumor mutational burden, neoantigens, tumor-infiltrating lymphocytes (TILs), T cell receptor clonality, and transcriptional signatures.

As the third most frequently mutated gene according to The Cancer Genome Atlas (TCGA), *MUC16* (OMIM 606154) encodes a membrane-spanning mucin, comprising a tandem repeat region sandwiched between N-terminal and C-terminal domains.^3,4^ Tumor antigen 125, residing in the tandem repeat region, is a common clinical biomarker to monitor epithelial ovarian cancer.^3^ A recent study^5^ reported that *MUC16* mutation may be associated with elevated tumor mutational burden and superior overall survival (OS) in patients with gastric adenocarcinoma. However, a comprehensive analysis of the relationship of *MUC16* mutation with ICI response across a large set of solid tumors is lacking. Here, we explored the associations of *MUC16* mutation with ICI response based on multidimensional data from multiple solid tumors.

## Methods

### Multidimensional Data Collection

This study was exempted from approval by an institutional review board and from the need for informed consent because its data were gathered from publicly available data sets that have received approval, in accordance with 45 CFR §46. This study follows the Strengthening the Reporting of Observational Studies in Epidemiology (STROBE) reporting guideline.

Somatic mutations, neoantigens, and gene expression data for TCGA samples from multiple solid tumor types were gathered. Specifically, somatic mutations of 10 195 samples and mRNA expression profiles of 9850 samples for 30 solid tumor types were downloaded from the TCGA Pan-Cancer Atlas project deposited in the cBioPortal database.^6,7^ These somatic mutations were uniformly called by the Multi-Center Mutation Calling in Multiple Cancers working group to adjust for variance and batch effects introduced by the differences in DNA extraction, hybridization-capture, sequencing, and analytical methods over time, which enables robust cross-tumor-type analyses.^8^ The mRNA expression levels were quantified using RNA-Seq by Expectation-Maximization algorithm^9^; batch-corrected for sequencers (Illumina GAII and HiSeq), sequencing centers (University of North Carolina and British Columbia Cancer Agency), and a plate effect identified in prostate adenocarcinoma; and normalized using the upper quartile method by Hoadley et al.^10^ Neoantigens of 5935 samples across 19 solid tumor types were obtained from The Cancer Immunome Atlas.^11^ Abundance data of 64 immune and stromal cell types in the tumor microenvironment for 9358 TCGA tumor samples were inferred using xCell^12^ and downloaded from the xCell website. xCell is a gene signatures–based method that uses a compensation technique to reduce spillover effects between closely related cell types. Overlapping associations of somatic mutations data with mRNA expression, neoantigens, and cell abundance data are shown in eFigure 1 in the Supplement.

### Clinical Cohorts Treated With ICIs

Uniformly analyzed clinical cohorts would help to more robustly evaluate biomarkers of response to ICI therapy.^13^ Thus, we obtained uniformly processed somatic mutations and clinical information of pretreatment tumors from patients with metastatic lung cancer or melanoma receiving ICI therapies. Briefly, all these patients were gathered from 5 published cohorts.^13,14,15,16,17^ To avoid the bias introduced by different studies, all raw whole-exome sequencing data (BAM files) and clinical annotations were processed through standardized quality control and mutation calling pipelines as described by Miao et al.^13^ We excluded 1 patient with small cell lung cancer from the lung cancer cohort with 57 patients and thus obtained 56 patients with non–small cell lung cancer (NSCLC) treated with anti-PD-1/PD-L1 as our NSCLC cohort. The melanoma cohort initially consisted of 151 patients treated with anti-CTLA-4, anti-PD-1, anti-PD-L1, or a combination of these therapies. Given the substantial differences in response rates and survival among patients treated with anti-CTLA-4, anti-PD-1, and anti-CTLA-4 plus anti-PD-1,^18^ we focused on 145 patients treated with anti-CTLA-4 alone to avoid potential bias introduced by different therapy classes.

With regard to the evaluation of clinical response to ICI therapy, several response definitions,^15,19,20^ which varied mostly in their assignment of patients with stable disease (SD) by Response Evaluation Criteria in Solid Tumors version 1.1,^21^ were reported. Given the evolving viewpoints on classifying clinical response to ICI therapy,^22^ a conservative method was applied: objective response was defined as complete response (CR) or partial response (PR) by Response Evaluation Criteria in Solid Tumors, and no response was defined as progressive disease (PD) by Response Evaluation Criteria in Solid Tumors.^13^

### *MUC16* Mutation and Tumor Mutational Burden

All nonsynonymous somatic mutations in *MUC16*, including missense, nonsense, nonstop, frame-shift insertion and deletion, in-frame insertion and deletion, and splice site mutations, were considered. Tumor mutational burden was calculated as the total count of nonsynonymous mutations in coding sequence using maftools.^23^

### Association of *MUC16* Mutation With Immune-Related Genes Expression Profile and Tumor Immune Microenvironment

First, we collected 40 immune-related genes, which were classified into 3 categories: immune checkpoint, T-effector and interferon-γ gene signature, and T cell receptor (eTable 1 in the Supplement). These genes were retrieved from previous reports.^24,25,26^ We next extracted normalized expression levels of these genes from 9580 samples with both somatic mutations and expression data available. The expression levels were log_2_-transformed after adding an offset of 1 to avoid taking log of 0 before analysis. In addition, we stratified all samples into 4 tumor immune microenvironment (TIME) types on the basis of median expression levels of *CD8A* (OMIM 186910) and *PD-L1* (OMIM 605402) according to the previous report,^27^ which defined the positive as gene expression above the median level. On the basis of 8755 samples with both somatic mutations and cell abundances data, we further evaluated the extent of immune cells infiltration into TIME by generating a composite immune score as the sum of abundances for B cells, CD4^+^ T cells, CD8^+^ T cells, dendritic cells, eosinophils, macrophages, monocytes, mast cells, neutrophils, and natural killer cells, as described elsewhere.^12^

### Survival Analysis

A Kaplan-Meier estimator of OS was used to construct the survival curves. The log-rank test was performed to compare OS of patients stratified by *MUC16* mutational status, and hazard ratios (HRs) with 95% CIs are provided. For the advanced melanoma cohort, complete clinical characteristics available included age at start of treatment and sex; 5 dominant COSMIC mutational signatures^28^ (signature 7, exposure to UV light; signature 5, unknown environmental exposure; signature 11, prior chemotherapeutic treatment with alkylating agents; signature 1, age-related spontaneous deamination of 5-methyl-cytosine; and signature 6, defective DNA mismatch repair) were also derived from the previous study,^13^ where mutational burden was not associated with ICI response after adjusting these dominant signatures, suggesting that it is a surrogate for an underlying mutagenic biological process. In melanoma, a large proportion of somatic mutations are known to arise from exposure to UV light.^29^ Finally, a Cox proportional hazards model was fitted for OS, controlling for potential confounding factors (age, sex, and mutational signatures, except signature 1) and ruling out multicollinearity. Multicollinearity among variables was determined by variance inflation factor calculated using the rms package in R statistical software version 3.5.2 (R Project for Statistical Computing). All survival analyses were performed using the survival package in R statistical software.

### Gene Set Enrichment Analysis

Gene set enrichment analysis (GSEA) was performed using the GSEA Desktop Application (version 3.0).^30,31^ Specifically, genes with 0 count in more than 40% of samples were first removed, and then log_2_-transformed fold changes for expression of qualified genes in *MUC16*-mutated vs wild-type states were calculated as a ranking metric for GSEAPreranked to perform GSEA against 50 hallmark gene sets annotated by the Molecular Signatures Database (version 6.2).^32^ This analysis involved 100 000 random permutations for gene set and weighted enrichment statistic. The normalized enrichment score was used as the magnitude of enrichment. The proportion of false-positives was controlled by calculating the false discovery rate (FDR). The FDR estimates the probability that a gene set with a given normalized enrichment score represents a false-positive finding. A significantly enriched gene set was expected at FDR < .001.

### Statistical Analysis

The Mann-Whitney *U* test was used to determine the association between *MUC16* mutation and a continuous variable. Fisher exact test and χ^2^ test were used to compare the frequencies of clinical response to ICIs and TIME types in *MUC16*-mutated vs wild-type tumors, respectively. The difference in mean mRNA expression of each immune-related gene in *MUC16*-mutated vs wild-type patients for different cancer types was hierarchically clustered with the pheatmap package in R statistical software, using Euclidean distance as a distance metric for samples, and ward.D2 clustering method. Statistical significance was expected at 2-tailed *P* < .05 unless otherwise specified. Statistical analyses were done in R statistical software version 3.5.2 (R Project for Statistical Computing). Data were obtained from October 1 through October 10, 2019, and were analyzed from October 11 through December 31, 2019.

## Results

### *MUC16* Mutation Associated With Elevated Tumor Mutational Burden and Neoantigen Load

Of the 10 195 patients with solid tumor from the TCGA Pan-Cancer Atlas project, 4821 (47.6%) were men, and the median (interquartile range [IQR]) age was 60 (50-70) years. The mutational frequency of *MUC16* was 19.68% (2006 of 10 195 patients). The frequency within each solid tumor type is summarized in eTable 2 in the Supplement, with the highest frequency, 73.86%, in skin cutaneous melanoma, followed by 42.76% in lung adenocarcinoma, and 38.84% in lung squamous cell carcinoma.

**Figure 1.**
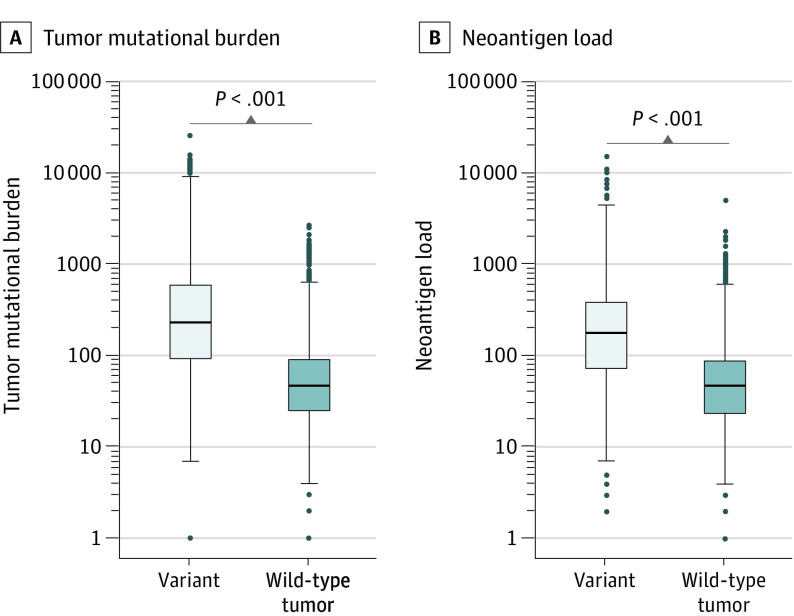
Association of *MUC16* Mutation With Tumor Mutational Burden and Neoantigen Load in Patients With Solid Tumor A and B, Graphs show comparison of tumor mutational burden (A) and neoantigen load (B) between *MUC16*-mutated and wild-type solid tumors from The Cancer Genome Atlas pan-cancer data set. Box plots show the median, first, and third quartiles; error bars extend to 1.5 times the interquartile range; and outlier data are shown as dots. Mann-Whitney *U* test was used for both comparisons.

To investigate the association of *MUC16* mutation with established genomic factors associated with ICI response, we began with tumor mutational burden, because it has been the most widely reproduced biomarker for ICI therapy.^33^ We found that, in this pan-cancer data set, patients with *MUC16* mutations exhibited significantly higher tumor mutational burden than those without them (median [IQR], 230 [93-595] mutations vs 48 [25-92] mutations; difference, 182 mutations; 95% CI, 164-199 mutations; *P* < .001, Mann-Whitney *U* test) (Figure 1A). This finding persisted within the majority of solid tumor types (25 of 30 tumor types) (eFigure 2 in the Supplement). We next examined the association of *MUC16* mutation with neoantigen load and observed that neoantigen load was significantly increased in patients with *MUC16*-mutated tumors vs those with wild-type tumors (median [IQR], 179 [74-394.5] neoantigens vs 48 [24-89] neoantigens; difference, 131 antigens; 95% CI, 116.5-145 neoantigens; *P* < .001, Mann-Whitney *U* test) (Figure 1B). Twelve of 19 solid tumor types had significantly increased load (eFigure 3 in the Supplement).

### *MUC16* Mutation Associated With Immune-Related Gene Signatures and Immune Infiltration

**Figure 2.**
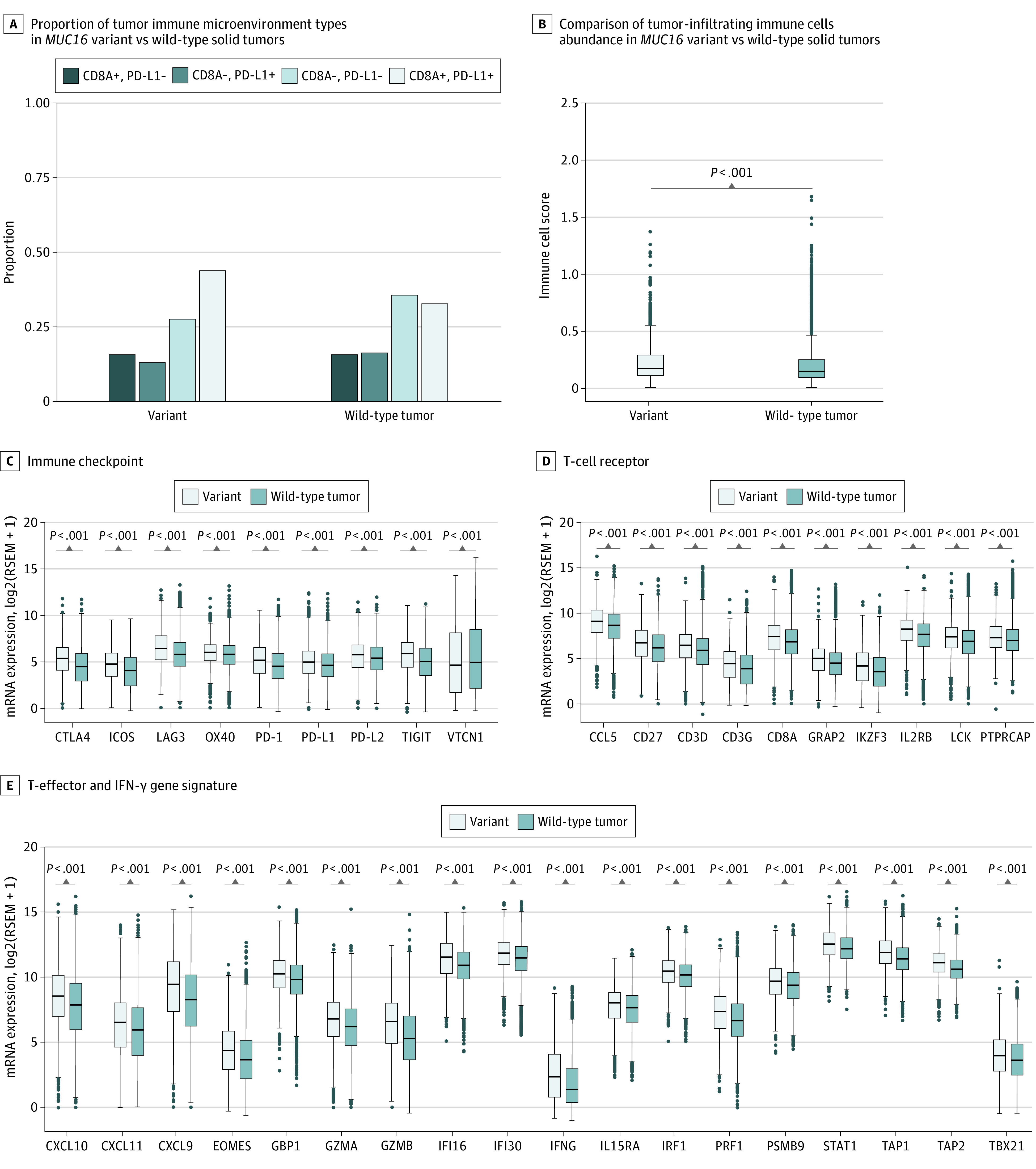
Association of *MUC16* Mutation With Immune Infiltration and Immune-Related Gene Signatures A, Proportion of tumor immune microenvironment (TIME) types in *MUC16*-mutated vs wild-type solid tumors. TIME I, 43.8% vs 32.4%; TIME II, 28.1% vs 36.3%; TIME III, 12.4% vs 16.1%; TIME IV, 15.7% vs 15.3%. B, Comparison of tumor-infiltrating immune cells abundance in *MUC16*-mutated vs wild-type solid tumors. C, D, and E, Differentially expressed genes classified into 3 categories, immune checkpoint (C), T-cell receptor (D), and T-effector and interferon-gamma gene signature (E). Box plots show the median, first, and third quartiles; error bars extend to 1.5 times the interquartile range; and outlier data are shown as dots. The χ^2^ test was used in A, and the Mann-Whitney *U* test was used in B, C, D, and E.

It has been proposed that 4 different types of TIMEs exist by measuring TILs recruitment, as evaluated by *CD8A* and *PD-L1* expression. Tumors that fit into TIME I, defined by positive *CD8A* and *PD-L1* expression, most likely receive benefit from anti-PD1/PD-L1 therapies.^27,34^ Thus, we categorized tumors into 4 groups according to median values of *CD8A* and *PD-L1* expression. We found that the proportion of TIME I (*CD8A* and *PD-L1 *positive) was significantly higher in *MUC16*-mutated tumors compared with wild-type ones (43.8% vs 32.4%; odds ratio, 1.63; 95% CI, 1.46-1.80; *P* < .001, χ^2^ test) (Figure 2A). To confirm the association between *MUC16* mutation and TIME, we generated a composite immune score based on immune cell types in innate and adaptive immune responses and observed a significantly elevated immune score in *MUC16*-mutated tumors compared with wild-type tumors (median [IQR] score, 0.17 [0.11-0.29] vs 0.14 [0.09-0.24]; difference, 0.03; 95% CI, 0.02-0.04; *P* < .001, Mann-Whitney *U* test) (Figure 2B), suggesting greater extent of immune cells infiltration into TIME. Interestingly, our analysis showed a significant positive correlation between *CD8A* expression and the composite immune score (Pearson correlation coefficient, 0.64; *P* < .001).

Given the link between *MUC16* mutation and TIME, we further investigated whether *MUC16* mutation was associated with immune-related gene signatures, including T-effector and interferon-γ gene signature, which has previously been associated with activated T cells, immune cytolytic activity, and interferon-γ expression.^35,36^ Of the 40 immune-related genes, 36 (90.0%) demonstrated higher expression levels in *MUC16*-mutated tumors in comparison with wild-type ones, but 1 gene (2.5%), *VTCN1* (OMIM 608162), showed decreased expression, implying a potential negative population for anti-VTCN1 immunotherapy (median [IQR] expression, 4.6 [1.7 to 8.1] vs 4.9 [2.1 to 8.5; difference, −0.3; 95% CI, −0.6 to 0.04; Bonferroni-corrected *P* < .05, Mann-Whitney *U* test) (Figure 2C, 2D, and 2E). The differences in mean expression levels of these genes in *MUC16*-mutated vs wild-type tumors from the respective cancer types are presented in eFigure 4 in the Supplement.

### *MUC16* Mutation Associated With Favorable Outcomes for ICI Treatment

**Figure 3.**
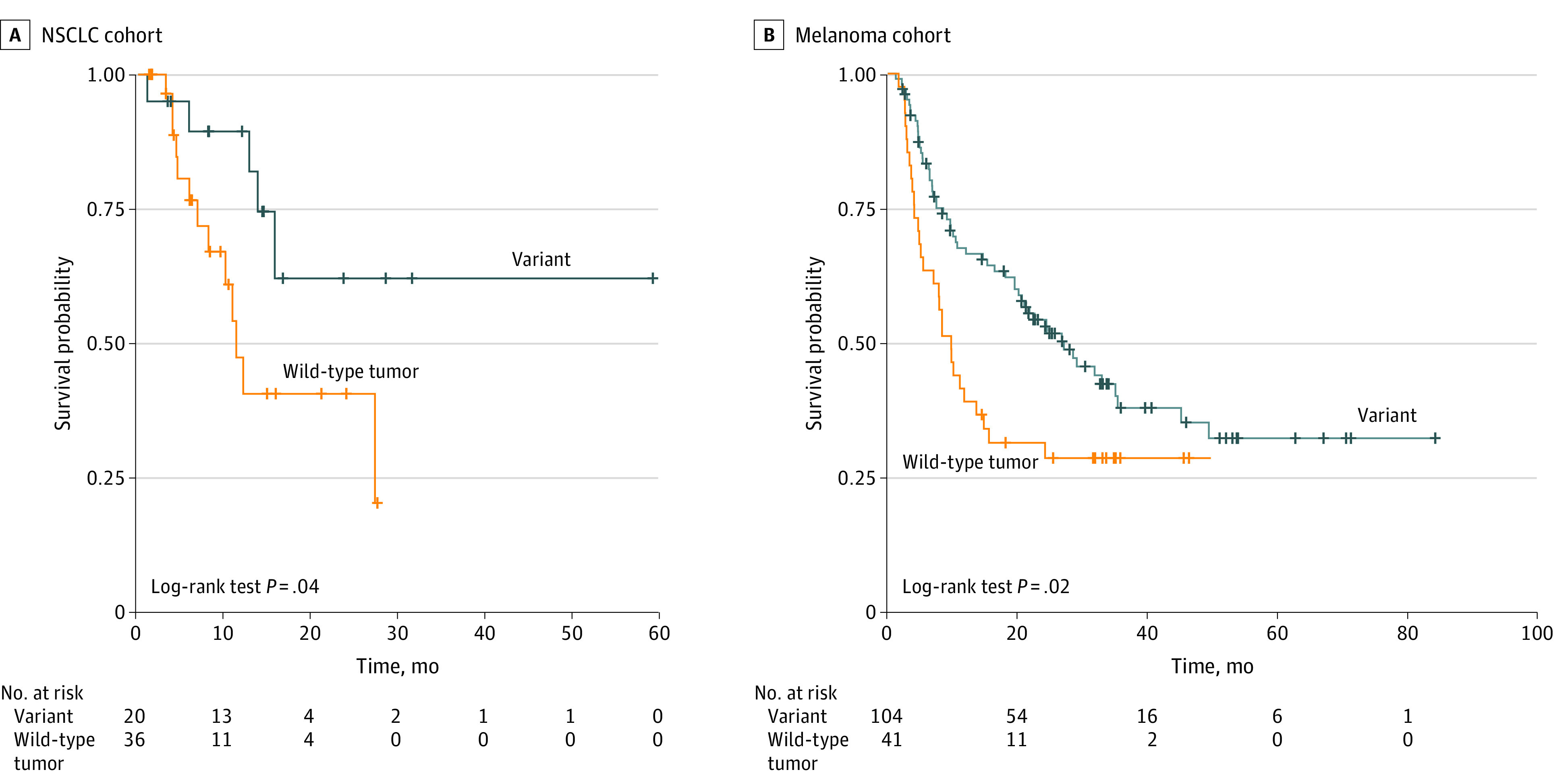
Overall Survival of Patients With *MUC16*-Mutated vs Wild-Type Tumors Treated With Immune Checkpoint Inhibitors A, Kaplan-Meier curves of overall survival in anti-PD-1/PD-L1–treated patients with non–small cell lung cancer (NSCLC). The median overall survival was 11.3 months (95% CI, 10.1 months to not available) in patients with wild-type tumors and was not reached in patients with *MUC16*-mutated tumors. B, Kaplan-Meier curves of overall survival in anti-CTLA4–treated patients with melanoma. The median overall survival was 27.03 months (95% CI, 20.07-45.00 months) vs 9.73 months (95% CI, 7.03-15.50 months) in patients with *MUC16*-mutated and wild-type tumors, respectively.

We further examined the association of *MUC16* mutation with outcomes in 2 ICI-treated cohorts, comprising 1 NSCLC cohort with 56 patients (median [IQR] age, 61 [57-66] years; 24 men [43%]) and 1 melanoma cohort with 145 patients (median [IQR] age, 61 [47-71] years; 98 men [67.6%]). As in the pan-cancer analyses, *MUC16* mutation was significantly associated with elevated tumor mutational burden (median mutational burden, NSCLC cohort, 325.0 vs 125.5 mutations; difference, 199.5 mutations; 95% CI, 49.0-290.0 mutations; *P* < .001, Mann-Whitney *U* test; melanoma cohort, 449.5 vs 82.0 mutations; difference, 367.5 mutations; 95% CI, 262.0-490.0 mutations; *P* < .001, Mann-Whitney *U* test) and neoantigen load (median neoantigen load, NSCLC cohort, 1038.0 vs 372.5 neoantigens; difference, 665.5 neoantigens; 95% CI, 100.9-920.7 neoantigens; *P* = .002, Mann-Whitney *U* test; melanoma cohort, 1404.5 vs 230.0; difference, 1174.5 neoantigens; 95% CI, 936.6-1614.5 neoantigens; *P* < .001, Mann-Whitney *U* test) in both cohorts (eFigure 5A and 5B and eFigure 6A and 6B in the Supplement). In Kaplan-Meier curves analysis for the NSCLC cohort, *MUC16* mutation was significantly associated with prolonged OS (median, not reached vs 11.3 months; hazard ratio, 0.34; 95% CI, 0.12-0.99; *P* = .04, log-rank test) (Figure 3A). There were 18 deaths in the NSCLC cohort.

**Figure 4.**
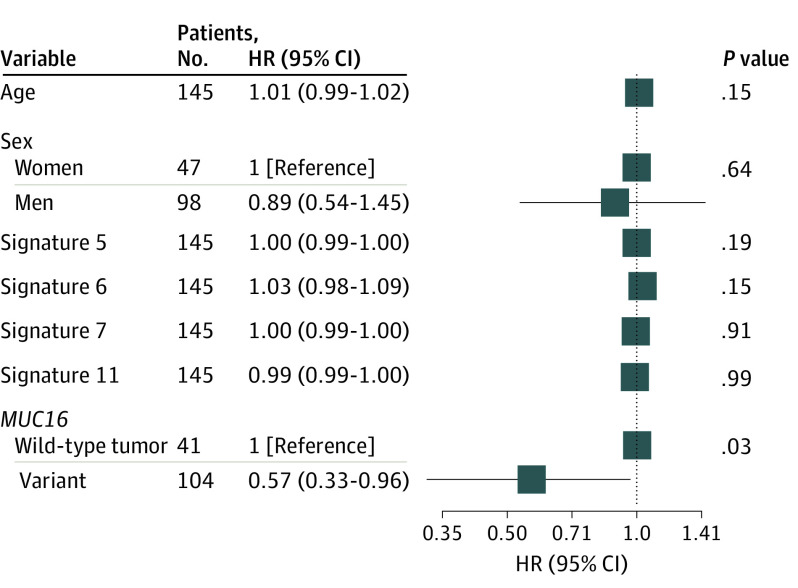
Forest Plot of Association Between *MUC16* Mutation and Overall Survival in Melanoma Cohort Data are adjusted for age, sex, and dominant mutational signatures. The vertical line represents hazard ratio (HR) of 1.0. Square data markers reflect estimated HRs. Error bars indicate 95% CIs.

We next confirmed the association between *MUC16* mutation and OS in the melanoma cohort. As shown in Figure 3B, significant OS improvement was also observed in patients with melanoma with *MUC16 *mutation (median, 27.03 vs 9.73 months; hazard ratio, 0.57; 95% CI, 0.36-0.90; *P* = .02, log-rank test). We used linear regression model to analyze the association of tumor mutational burden with neoantigen load and observed that each nonsynonymous mutation produced, on average, 2.79 estimated neoantigens, with a strong correlation between them (*R*^2^ = 0.98; *P* < 2 × 10^−16^) (eFigure 7 in the Supplement), making it difficult to extricate the association of each burden on response from the other. On the basis of the mutational signature activity in each patient with melanoma (eFigure 8 in the Supplement), we observed significant associations of UV light–related signature 7 (median contribution, 0.71 vs 0.15; difference, 0.56; 95% CI, 0.3-0.68; *P* < .001, Mann-Whitney *U* test) and alkylating agents-related signature 11 (median contribution, 0.21 vs 0.11; difference, 0.10; 95% CI, 0.02-0.19; *P* < .001, Mann-Whitney *U* test) with *MUC16* mutation (eFigure 9 in the Supplement). To eliminate the possibility that the association was skewed by confounders, a multivariable Cox model was adopted to adjust for age, sex, and dominant mutational signatures. No multicollinearity was detected in this model, whereas high variance inflation factor was observed for tumor mutational burden when including it (variance inflation factor = 5.24) (eTable 3 in the Supplement). The significant association persisted after controlling these factors (hazard ratio, 0.57; 95% CI, 0.33-0.96; *P* = .04) (Figure 4).

**Figure 5.**
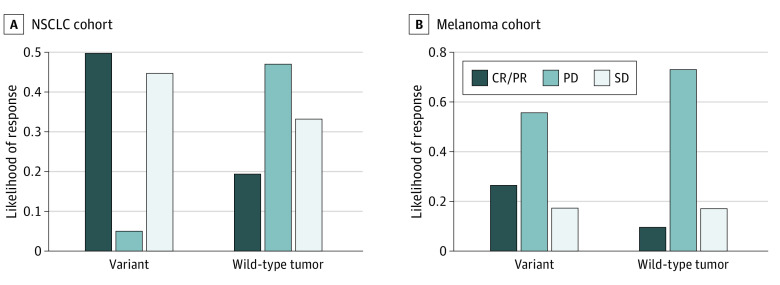
Comparison of Response Rates to Immune Checkpoint Therapy Between Patients With *MUC16*-Mutated and Wild-Type Tumors A and B, Likelihood of response stratified by *MUC16* mutational status in anti-PD-1/PD-L1–treated patients with non–small cell lung cancer (NSCLC) (A) and anti-CTLA4–treated patients with melanoma (B). CR/PR indicates complete response or partial response; PD, progressive disease; SD, stable disease.

We also examined the likelihood of response to ICI therapy stratified by *MUC16* mutational status and found that *MUC16* mutation was enriched in patients with CR or PR for both the NSCLC cohort (10 of 20 patients with *MUC16* mutation [50%] vs 7 of 36 patients with wild-type tumor [19%]; odds ratio, 4.03; 95% CI, 1.06-16.43; *P* = .03, Fisher exact test) and melanoma cohort (28 of 104 patients with *MUC16 *mutation [27%] vs 4 of 41 patients with wild-type tumor [10%]; odds ratio, 3.38; 95% CI, 1.07-14.25; *P* = .03, Fisher exact test) (Figure 5A and 5B).

### Enriched Biological Processes in *MUC16*-Mutated Tumors

To identify biological processes associated with *MUC16* mutational status, we conducted GSEA on 50 hallmark gene sets, representing major biological processes, for 9580 patients with and without *MUC16* mutations. Our analysis yielded 8 gene sets whose expression was significantly upregulated in patients with *MUC16* mutations (FDR < .001) (eFigure 10 in the Supplement). Upon inspecting these gene sets, we were able to assign them to 3 biological themes involved in cell proliferation (E2F targets, G2/M checkpoint, MYC targets variant 1, and MYC targets variant 2), immune response (interferon-α response, interferon-γ response, and allograft rejection), and mTORC1 signaling.

## Discussion

Our analysis of the TCGA pan-cancer data set across multiple solid tumor types demonstrated that *MUC16* mutation was associated with factors previously associated with response to ICI therapy. For instance, patients with *MUC16* mutations exhibited higher tumor mutational burden and neoantigen load, compared with patients with wild-type tumor, indicating increased tumor immunogenicity. In a recent study^5^ of gastric adenocarcinoma, *MUC16* mutation was associated with elevated tumor mutational burden. On the basis of a simplistic and pragmatic framework of stratifying tumors, we classified all patients into 4 different TIMEs (I, *PD-L1* positive with TILs driving adaptive immune resistance; II, *PD-L1* negative with no TIL indicating immune ignorance; III, *PD-L1* positive with no TIL indicating intrinsic induction; and IV, *PD-L1* negative with TILs indicating the role of other suppressors in promoting immune tolerance)^34^ and found a higher proportion of TIME I in patients with *MUC16* mutation. Notably, TIME I was reported to be associated with a high mutational burden, abundant neoantigen, *PD-L1* amplification, and infection with an oncogenic virus, representing an immunoresponsive microenvironment to anti-PD1/PD-L1 therapies.^2^ This finding is further supported by *MUC16*-mutated tumors characterized by upregulated expression of T-effector and interferon-γ gene signature, a hallmark of preexisting immunity associated with pronounced benefit from checkpoint blockade.^36^ Furthermore, the greater abundance of immune cells observed in the microenvironment of *MUC16*-mutated tumors might more directly demonstrate the association of *MUC16* mutation with TIME. An additional hallmark of *MUC16*-mutated tumors is the augmented expression of multiple inhibitory checkpoints, such as *PD-L1*, *PD-1*, *CTLA4*, *LAG3*, and others, suggesting that there exists potential adaptive immune resistance to anti-PD-1/PD-L1 therapies and that additional inhibitory pathways beyond the PD-1/PD-L1 axis might be targetable.^37^ Taken together, these findings support the hypothesis that *MUC16* mutation is associated with high immunogenicity and a responsive TIME with PD-L1–dependent or PD-L1–independent adaptive immune resistance.^38^ Consistent with the hypothesis, to our knowledge, we first confirmed the association of *MUC16* mutation with superior outcomes in cohorts receiving ICI treatment. *MUC16* mutation was associated with improved OS and response rates in anti-PD-1/PD-L1–treated patients with NSCLC, and this finding was verified in the independent melanoma cohort treated with anti-CTLA-4.

*MUC16* has previously been implicated in tumor cell proliferation, metastasis, and modulation of the innate immune response by direct suppression of natural killer cell function.^3,4^ Also, our GSEA analysis revealed that 8 biological processes regarding cell proliferation, immune response, and mTORC1 signaling were significantly upregulated in *MUC16*-mutated tumors, providing biological insights concerning the link between *MUC16* mutation and ICI response.

### Limitations

Our study has several limitations. First, although *MUC16*-mutated solid tumors were characterized by alteration of pathways involved in cell proliferation, immune response, and mTORC1 signaling, mechanistic underpinnings of *MUC16* mutation relevant to ICI response remain elusive and merit further experimental work. Second, the sample size of both ICI-treated cohorts was limited. The limited number of deaths (18 deaths) in the NSCLC cohort restricted the ability to controlling confounders. Additional and larger clinical studies are required. Despite these limitations, this observed association between *MUC16* mutation and ICI response in this pan-cancer study represents a further step toward the development of factors associated with outcomes for ICI therapy in solid tumors.

## Conclusions

*MUC16* mutation is associated with established genomic factors associated with response and better outcomes for ICI therapy in solid tumors. It may serve as a prognostic stratification factor to help optimize the application of ICI-based immunotherapy.

## Notes

Supplement. **eTable 1. **Summary of the 40 Immune-Related Genes **eTable 2. **Prevalence of *MUC16* Mutations Across 30 Solid Tumor Types **eTable 3.** Assessment of Multicollinearity for the Cox Model With and Without Tumor Mutational Burden in Melanoma Cohort **eFigure 1. **Venn Diagrams of Number of Samples With Each Type of Data Available **eFigure 2. **Correlation of *MUC16* Mutation With Tumor Mutational Burden Across 30 Solid Tumor Types **eFigure 3. **Correlation of *MUC16* Mutation With Neoantigen Load Across 19 Solid Tumor Types **eFigure 4. **Unsupervised Hierarchical Clustering of 37 Differentially Expressed Immune-Related Genes Across 30 Solid Tumor Types **eFigure 5.** Correlation of *MUC16* Mutation With Tumor Mutational Burden and Neoantigen Load in Non-small Cell Lung Cancer Cohort **eFigure 6.** Correlation of *MUC16* Mutation With Tumor Mutational Burden and Neoantigen Load in Melanoma Cohort **eFigure 7.** Relationship Between Tumor Mutational Burden and Neoantigen Load in Melanoma Cohort **eFigure 8.** Mutational Signature Activity in Melanoma Cohort **eFigure 9.** Correlation of *MUC16* Mutation With Ultraviolet Light or Alkylating Agents-Related Signatures **eFigure 10. **Gene Set Enrichment Analysis Plots of Significantly Enriched Gene Sets
