# Supplementary material for: Association of MUC16 Mutation With Response to Immune Checkpoint Inhibitors in Solid Tumors
Source: JAMA Netw Open. 2020 Aug 26;3(8):e2013201. doi: 10.1001/jamanetworkopen.2020.13201 (PMC7450349; doi:10.1001/jamanetworkopen.2020.13201)
Supplement: Supplement. — eTable 1. Summary of the 40 Immune-Related Genes eTable 2. Prevalence of MUC16 Mutations Across 30 Solid Tumor Types eTable 3. Assessment of Multicollinearity for the Cox Model With and Without Tumor Mutational Burden in Melanoma Cohort eFigure 1. Venn Diagrams of Number of Samples With Each Type of Data Available eFigure 2. Correlation of MUC16 Mutation With Tumor Mutational Burden Across 30 Solid Tumor Types eFigure 3. Correlation of MUC16 Mutation With Neoantigen Load Across 19 Solid Tumor Types eFigure 4. Unsupervised Hierarchical Clustering of 37 Differentially Expressed Immune-Related Genes Across 30 Solid Tumor Types eFigure 5. Correlation of MUC16 Mutation With Tumor Mutational Burden and Neoantigen Load in Non-small Cell Lung Cancer Cohort eFigure 6. Correlation of MUC16 Mutation With Tumor Mutational Burden and Neoantigen Load in Melanoma Cohort eFigure 7. Relationship Between Tumor Mutational Burden and Neoantigen Load in Melanoma Cohort eFigure 8. Mutational Signature Activity in Melanoma Cohort eFigure 9. Correlation of MUC16 Mutation With Ultraviolet Light or Alkylating Agents-Related Signatures eFigure 10. Gene Set Enrichment Analysis Plots of Significantly Enriched Gene Sets [file jamanetwopen-3-e2013201-s001.pdf]

Supplementary Online Content

Zhang L, Han X, Shi Y. Association of *MUC16* mutation with response to immune checkpoint inhibitors in solid tumors. *JAMA Netw Open*. 2020;3(8):e2013201. doi:10.1001/jamanetworkopen.2020.13201

eTable 1. Summary of the 40 Immune-Related Genes
eTable 2. Prevalence of *MUC16* Mutations Across 30 Solid Tumor Types
eTable 3. Assessment of Multicollinearity for the Cox Model With and Without Tumor Mutational Burden in Melanoma Cohort
eFigure 1. Venn Diagrams of Number of Samples With Each Type of Data Available
eFigure 2. Correlation of *MUC16* Mutation With Tumor Mutational Burden Across 30 Solid Tumor Types
eFigure 3. Correlation of *MUC16* Mutation With Neoantigen Load Across 19 Solid Tumor Types
eFigure 4. Unsupervised Hierarchical Clustering of 37 Differentially Expressed Immune-Related Genes Across 30 Solid Tumor Types
eFigure 5. Correlation of *MUC16* Mutation With Tumor Mutational Burden and Neoantigen Load in Non-small Cell Lung Cancer Cohort
eFigure 6. Correlation of *MUC16* Mutation With Tumor Mutational Burden and Neoantigen Load in Melanoma Cohort
eFigure 7. Relationship Between Tumor Mutational Burden and Neoantigen Load in Melanoma Cohort
eFigure 8. Mutational Signature Activity in Melanoma Cohort
eFigure 9. Correlation of *MUC16* Mutation With Ultraviolet Light or Alkylating Agents-Related Signatures
eFigure 10. Gene Set Enrichment Analysis Plots of Significantly Enriched Gene Sets

This supplementary material has been provided by the authors to give readers additional information about their work.

© 2020 Zhang L et al. *JAMA Network Open*.

eTable 1. Summary of the 40 Immune-Related Genes

Category	Genes
Immune checkpoint	*PD-1* (OMIM 600244), *CTLA4* (OMIM 123890), *LAG3* (OMIM 153337), *TIM3* (OMIM 606652), *ICOS* (OMIM 604558), *OX40* (OMIM 600315), *PD-L1* (OMIM 605402), *PD-L2* (OMIM 605723), *VTCN1* (OMIM 608162), *TIGIT* (OMIM 612859)
T-effector and interferon-gamma gene signature	*GBP1* (OMIM 600411), *IFI16* (OMIM 147586), *IFI30* (OMIM 604664), *IRF1* (OMIM 147575), *STAT1* (OMIM 600555), *TAP1* (OMIM 170260), *TAP2* (OMIM 170261), *PSMB9* (OMIM 177045), *IL15RA* (OMIM 601070), *GZMA* (OMIM 140050), *GZMB* (OMIM 123910), *IFNG* (OMIM 147570), *EOMES* (OMIM 604615), *CXCL9* (OMIM 601704), *CXCL10* (OMIM 147310), *TBX21* (OMIM 604895), *CXCL11* (OMIM 604852), *PRF1* (OMIM 170280)
T cell receptor	*CD27* (OMIM 186711), *GRAP2* (OMIM 604518), *LCK* (OMIM 153390), *PTPRCAP* (OMIM 601577), *CCL5* (OMIM 187011), *IL2RB* (OMIM 146710), *IKZF3* (OMIM 606221), *CD3G* (OMIM 186740), *CD74* (OMIM 142790), *CD3D* (OMIM 186790), *CD8A* (OMIM 186910), *CD4* (OMIM 186940)

© 2020 Zhang L et al. *JAMA Network Open*.

eTable 2. Prevalence of *MUC16* Mutations Across 30 Solid Tumor Types

Disease Name	Disease Abbreviation	No. of Total Samples	No. of Affected Samples	Percentage of Affected Samples
Adrenocortical carcinoma	ACC	91	14	15.38%
Bladder urothelial carcinoma	BLCA	410	119	29.02%
Breast invasive carcinoma	BRCA	1066	109	10.23%
Cervical and endocervical cancers	CESC	291	48	16.49%
Cholangiocarcinoma	CHOL	36	3	8.33%
Colorectal adenocarcinoma	COADREAD	534	146	27.34%
Esophageal carcinoma	ESCA	182	43	23.63%
Glioblastoma multiforme	GBM	397	61	15.37%
Head and Neck squamous cell carcinoma	HNSC	515	101	19.61%
Kidney Chromophobe	KICH	65	3	4.62%
Kidney renal clear cell carcinoma	KIRC	402	30	7.46%
Kidney renal papillary cell carcinoma	KIRP	276	27	9.78%
Brain Lower Grade Glioma	LGG	512	36	7.03%
Liver hepatocellular carcinoma	LIHC	366	60	16.39%
Lung adenocarcinoma	LUAD	566	242	42.76%

© 2020 Zhang L et al. *JAMA Network Open*.

Lung squamous cell carcinoma	LUSC	484	188	38.84%
Mesothelioma	MESO	86	1	1.16%
Ovarian serous cystadenocarcinoma	OV	523	47	8.99%
Pancreatic adenocarcinoma	PAAD	179	13	7.26%
Pheochromocytoma and Paraganglioma	PCPG	178	5	2.81%
Prostate adenocarcinoma	PRAD	494	31	6.28%
Sarcoma	SARC	254	33	12.99%
Skin Cutaneous Melanoma	SKCM	440	325	73.86%
Stomach adenocarcinoma	STAD	436	146	33.49%
Testicular Germ Cell Tumors	TGCT	145	2	1.38%
Thyroid carcinoma	THCA	490	13	2.65%
Thymoma	THYM	123	8	6.50%
Uterine Corpus Endometrial Carcinoma	UCEC	517	142	27.47%
Uterine Carcinosarcoma	UCS	57	6	10.53%
Uveal Melanoma	UVM	80	4	5.00%

© 2020 Zhang L et al. *JAMA Network Open*.

eTable 3. Assessment of Multicollinearity for the Cox Model With and Without Tumor Mutational Burden in Melanoma Cohort

	VIF	
	**Cox model with TMB[a]**	**Cox model without TMB**
Age	1.24	1.36
Sex	1.23	1.24
Signature 5	1.2	1.21
Signature 6	1.33	1.17
Signature 7	2.25	1.19
Signature 11	1.98	1.29
MUC16	2.1	1.11
TMB	5.24	NA

Abbreviation: VIF, variance inflation factor; TMB, tumor mutational burden; NA, not applicable.

[a]Log_2-transformed TMB was entered in the model.

© 2020 Zhang L et al. *JAMA Network Open*.

eFigure 1. Venn Diagrams of Number of Samples With Each Type of Data Available

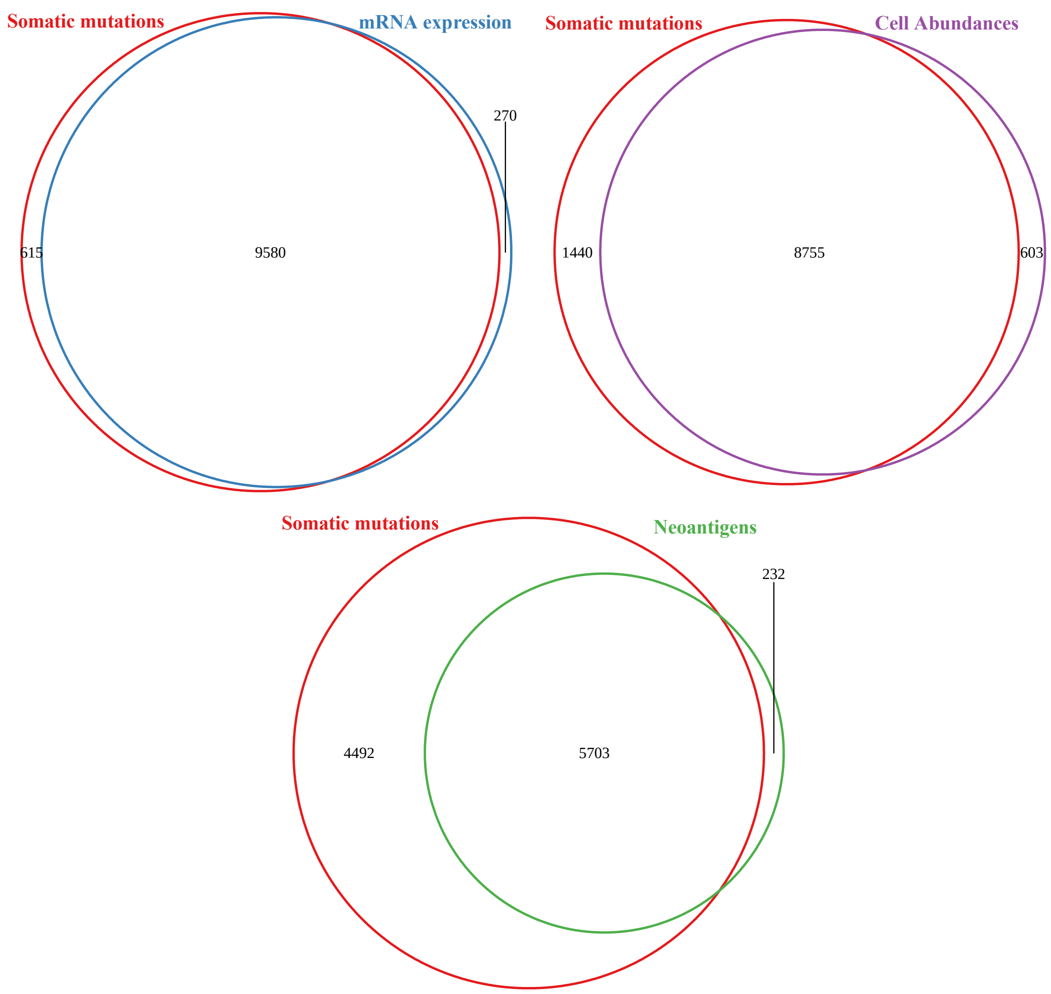

© 2020 Zhang L et al. *JAMA Network Open*.

eFigure 2. Correlation of *MUC16* Mutation With Tumor Mutational Burden Across 30 Solid Tumor Types

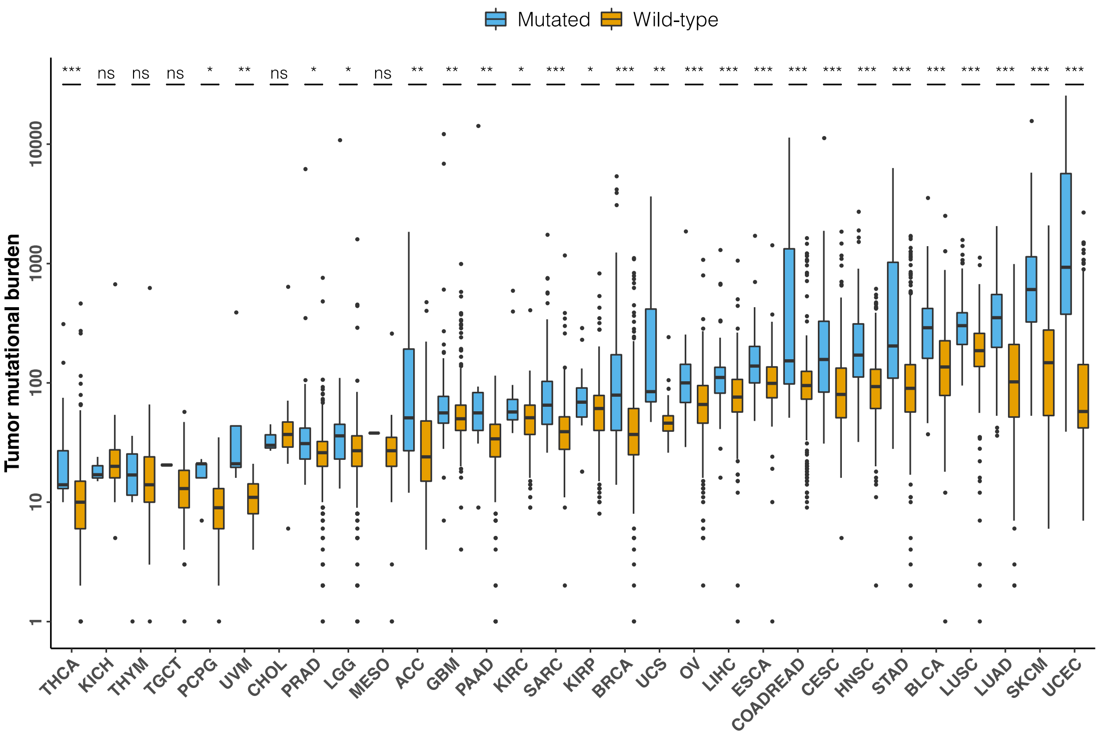

Box plots depict the comparison of tumor mutational burden in *MUC16*-mutated versus wild-type cancers. The horizontal axis denotes the respective cancer types ordered on the basis of the median tumor mutational burden of *MUC16*-mutated cancer subgroup. Mann-Whitney *U* test was used for all comparisons: *, *P* < .05, **, *P* < .01, ***, *P* < .001, ns, not significant. Box plots show the first, median, and third quartiles, whiskers extend to 1.5 times the interquartile range, and outlier data are shown as dots.

© 2020 Zhang L et al. *JAMA Network Open*.

eFigure 3. Correlation of *MUC16* Mutation With Neoantigen Load Across 19 Solid Tumor Types

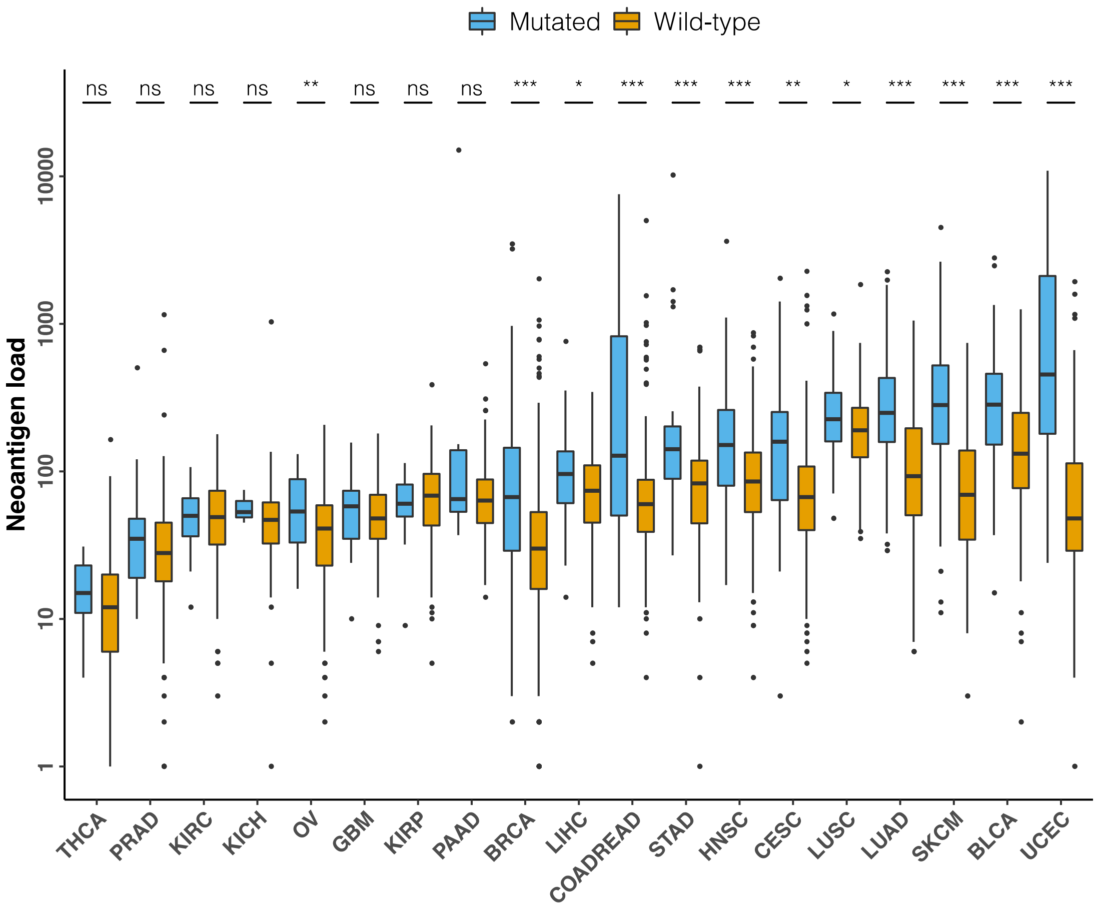

Box plots depict the comparison of neoantigen load in *MUC16*-mutated versus wild-type cancers. The horizontal axis denotes the respective cancer types ordered according to the median neoantigen load of *MUC16*-mutated cancer subgroup. Mann-Whitney *U* test was used for all comparisons: *, $P < .05$, **, $P < .01$, ***, $P < .001$, ns, not significant. Box plots show the first, median, and third quartiles, whiskers extend to 1.5 times the interquartile range, and outlier data are shown as dots.

© 2020 Zhang L et al. *JAMA Network Open*.

eFigure 4. Unsupervised Hierarchical Clustering of 37 Differentially Expressed Immune-Related Genes Across 30 Solid Tumor Types

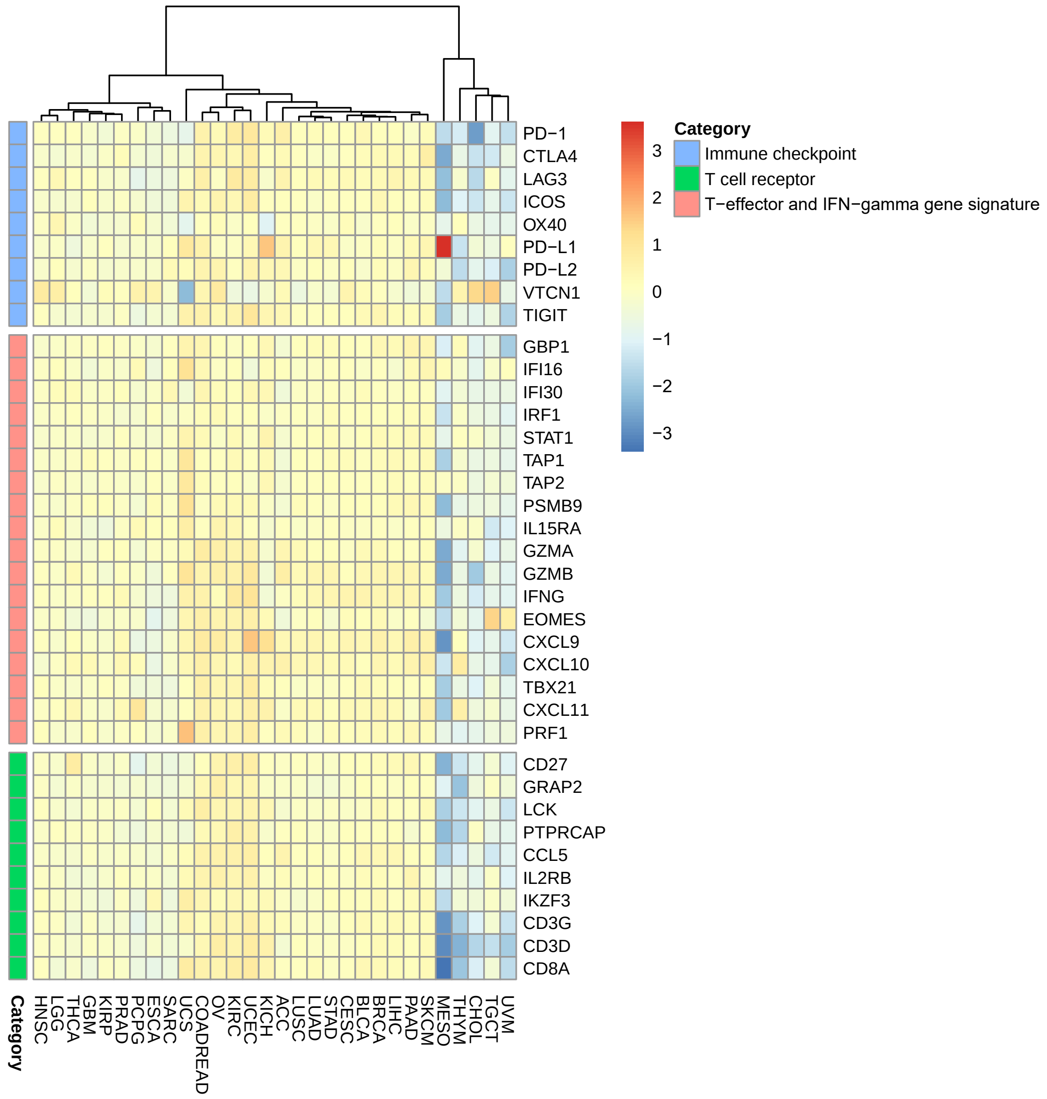

The row denotes 37 differentially expressed immune-related genes classified into immune checkpoint, T-effector and interferon (IFN) -gamma gene signature, and T cell receptor categories whereas the different cancer types are on the column. Shown in each square is the difference of mean mRNA expression level of each gene in the corresponding *MUC16*-mutated versus wild-type cancer. Specifically, the difference is calculated by subtracting the gene's mean expression level of wild-type patients from its mean expression level of *MUC16*-mutated patients in one cancer type, then the differences are depicted in colors from dark blue (higher expression in the wild-type cancer), to dark red (higher expression in the *MUC16*-mutated cancer) as shown in the color bar.

© 2020 Zhang L et al. *JAMA Network Open*.

eFigure 5. Correlation of *MUC16* Mutation With Tumor Mutational Burden and Neoantigen Load in Non-small Cell Lung Cancer Cohort

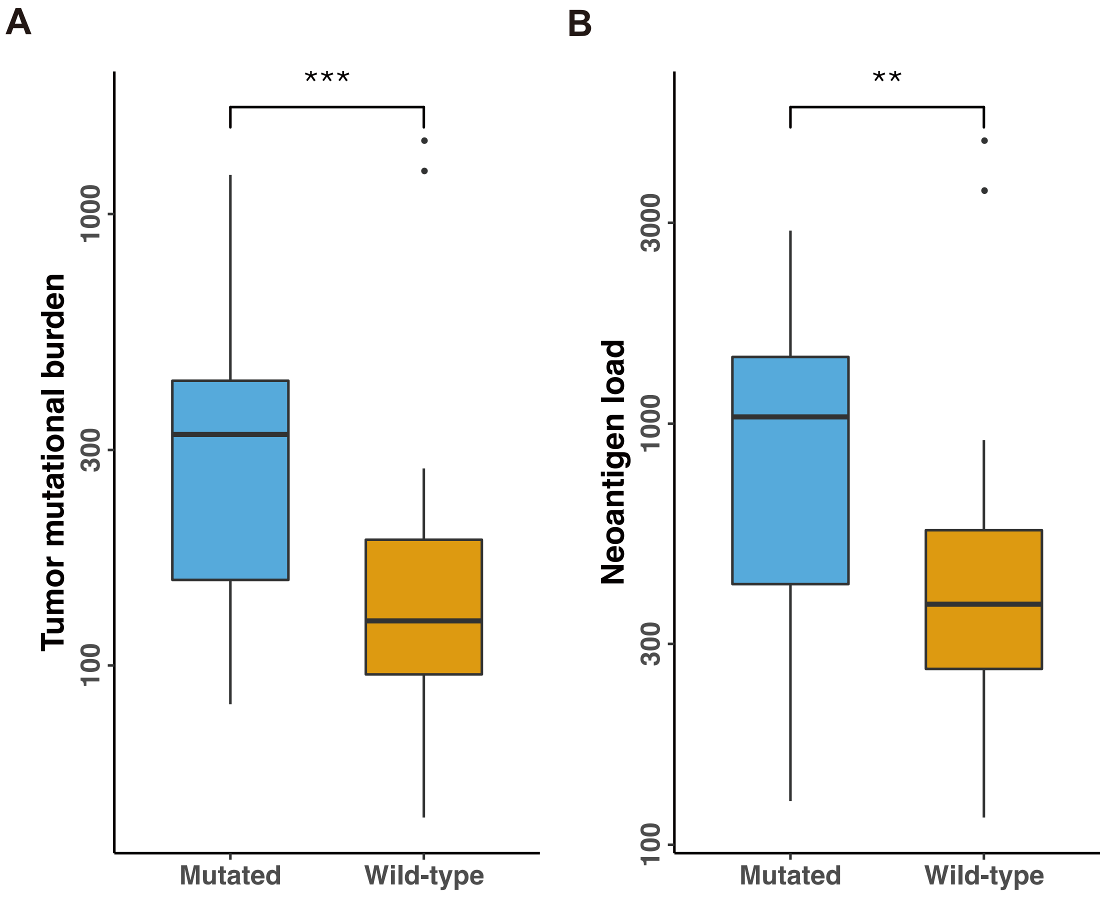

A and B, Comparison of tumor mutational burden (A) and neoantigen load (B) between *MUC16*-muatated and wild-type solid cancers from the non-small cell lung cancer cohort, respectively. Mann-Whitney *U* test was used in A and B: **, *P* < .01; ***, *P* < .001. Box plots show the median, first, and third quartiles, whiskers extend to 1.5 times the interquartile range, and outlier data are shown as dots.

© 2020 Zhang L et al. *JAMA Network Open*.

eFigure 6. Correlation of *MUC16* Mutation With Tumor Mutational Burden and Neoantigen Load in Melanoma Cohort

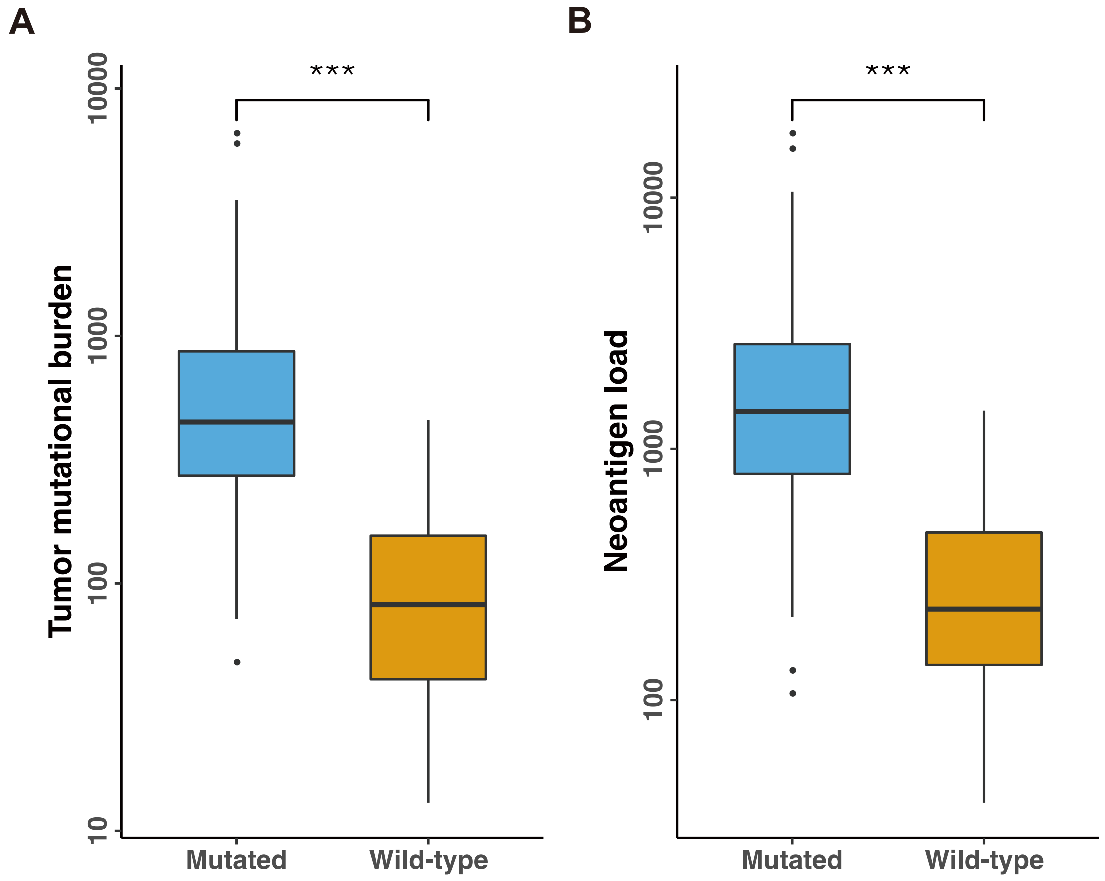

A and B, Comparison of tumor mutational burden (A) and neoantigen load (B) between *MUC16*-muatated and wild-type solid cancers from the melanoma cohort, respectively. Mann-Whitney *U* test was used in A and B: ***, $P < .001$. Box plots show the median, first, and third quartiles, whiskers extend to 1.5 times the interquartile range, and outlier data are shown as dots.

© 2020 Zhang L et al. *JAMA Network Open*.

eFigure 7. Relationship Between Tumor Mutational Burden and Neoantigen Load in Melanoma Cohort

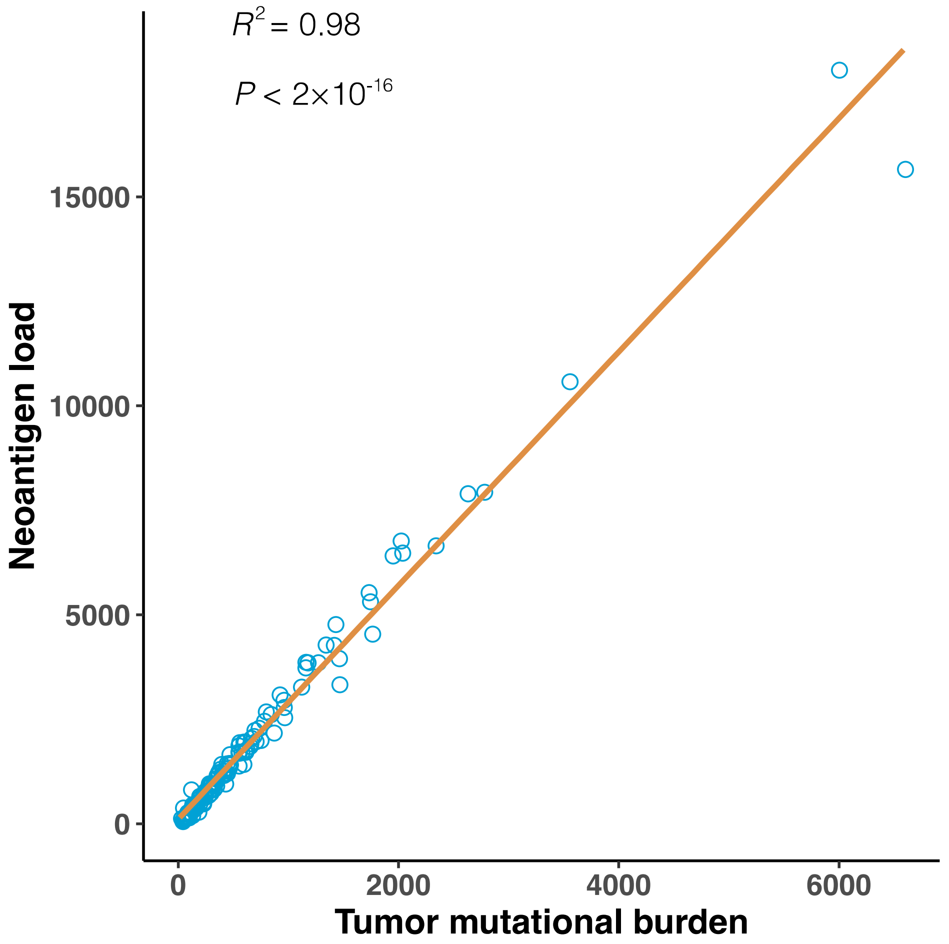

The linear regression formula for neoantigen load is 2.79 × (tumor mutational burden) + 112.26 in the melanoma cohort.

© 2020 Zhang L et al. *JAMA Network Open*.

eFigure 8 Mutational Signature Activity in Melanoma Cohort

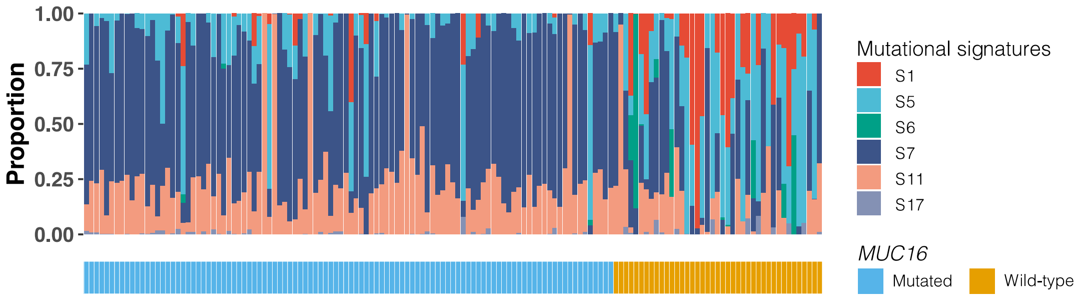

The filled bars depict the mutational signature activities of 145 patients stratified by *MUC16* mutational status in the melanoma cohort. The vertical axis denotes the proportion of mutations attributable to each mutational signature (S1, S5, S6, S7, S11, and S17) in each patient.

© 2020 Zhang L et al. *JAMA Network Open*.

eFigure 9 Correlation of *MUC16* Mutation With Ultraviolet Light or Alkylating Agents-Related Signatures

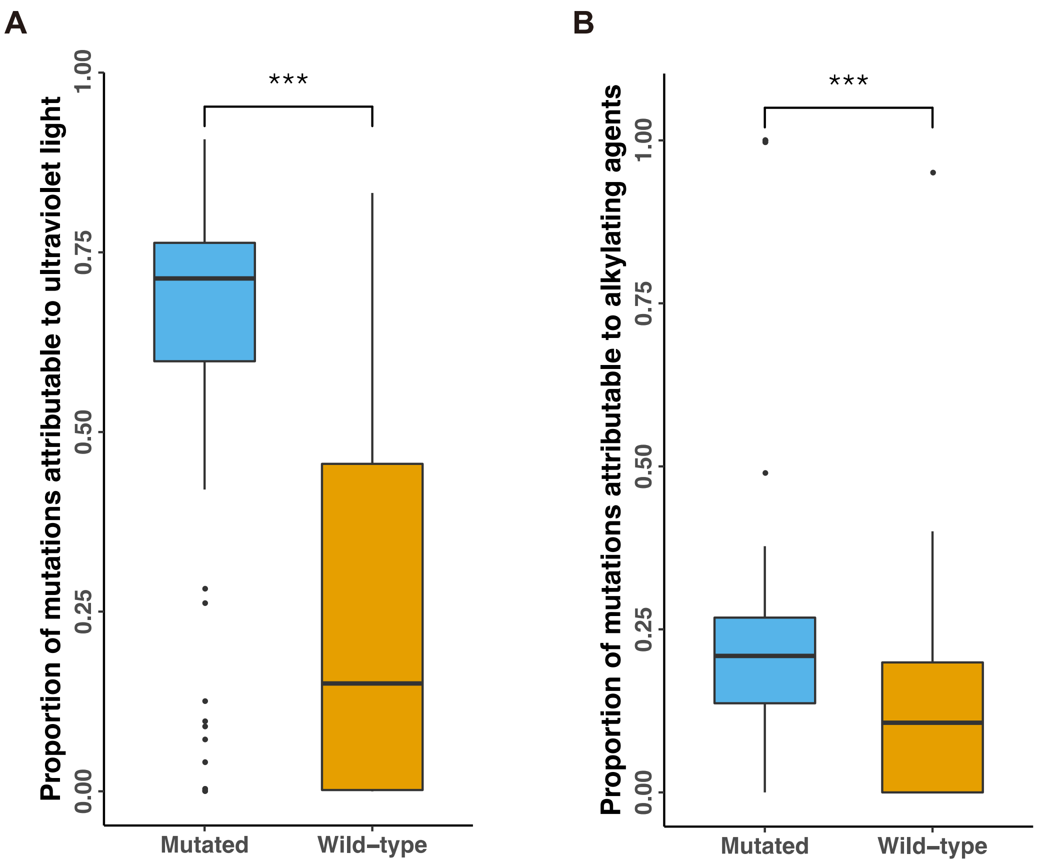

A and B, Comparison of the proportion of mutations attributable to ultraviolet light-related signatures 7 (A) and alkylating agents-related signatures 11 (B) between *MUC16*-muatated and wild-type solid cancers from the melanoma cohort, respectively. Mann-Whitney *U* test was used in A and B: ***, *P* < .001. Box plots show the median, first, and third quartiles, whiskers extend to 1.5 times the interquartile range, and outlier data are shown as dots.

© 2020 Zhang L et al. *JAMA Network Open*.

eFigure 10. Gene Set Enrichment Analysis Plots of Significantly Enriched Gene Sets

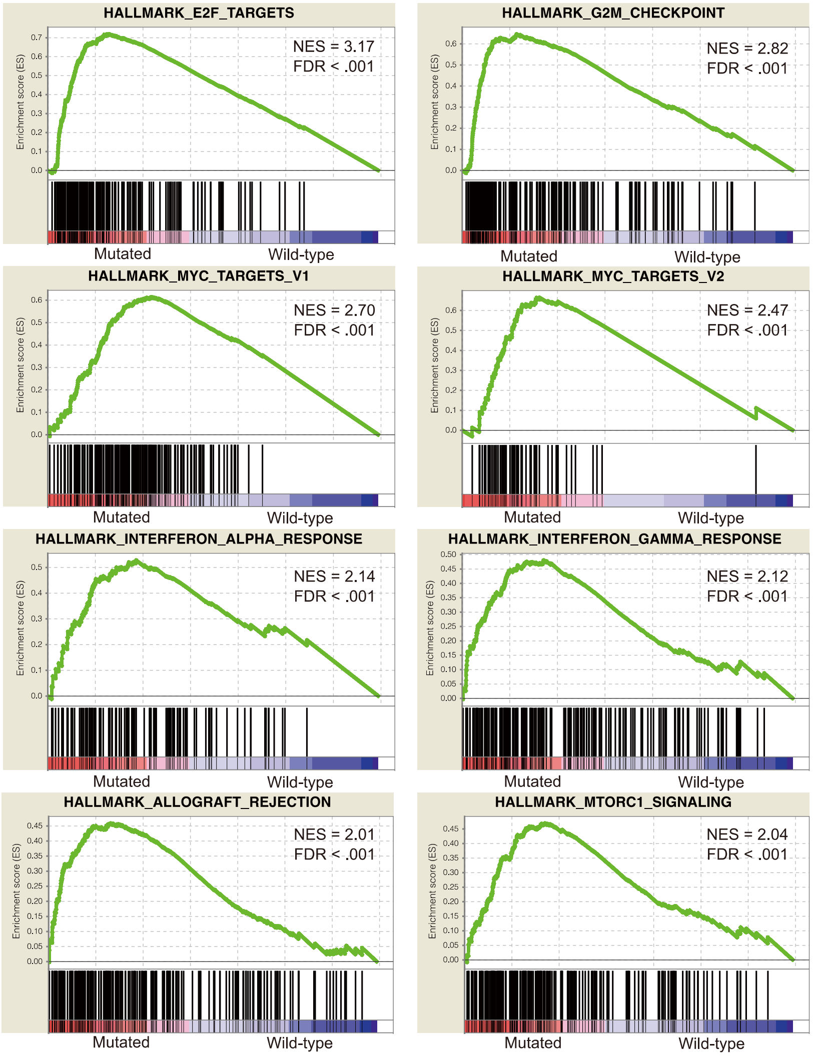

Gene set enrichment analysis is applied to identify eight hallmark gene sets whose expression is positively correlated with *MUC16* mutation (All FDR < .001). These included E2F TARGETS which comprises genes encoding cell cycle related targets of E2F transcription factors, G2M CHECKPOINT which comprises genes involved in the G2/M checkpoint, MYC TARGETS V1 which comprises a subgroup of genes regulated by MYC - version 1, MYC TARGETS V2 which comprises a subgroup of genes regulated by MYC - version 2, INTERFERON ALPHA RESPONSE which comprises genes upregulated in response to alpha interferon proteins, INTERFERON GAMMA RESPONSE which comprises genes upregulated in response to IFNG, ALLOGRAFT REJECTION which comprises genes upregulated during transplant rejection, and MTORC1 SIGNALING which comprises genes upregulated through activation of mTORC1 complex. NES, normalized enrichment score.

© 2020 Zhang L et al. *JAMA Network Open*.
